# The Reach of the Gastrocnemius Musculocutaneous Flap: How High Is High?

**DOI:** 10.29252/wjps.7.3.319

**Published:** 2018-09

**Authors:** Nikhil Panse, Rahul Bhadgale, Ankur Karanjkar, Rohit Phulwer, Parag Sahasrabudhe, Chaitanya Ramteke

**Affiliations:** Department of Plastic Surgery, BJG Medical College and Sassoon Hospital, Pune, India

**Keywords:** Gastrocnemius muscle, Musculocutaneous, Thigh, Flap, Defect

## Abstract

**BACKGROUND:**

Gastrocnemius muscle and musculocutaneous flaps are very versatile and one of the commonly used flaps for lower extremity reconstruction. There is significant literature available on the use of these flaps. However, we feel that the potential of the gastrocnemius musculocutaneous (GMC) flaps has not yet been fully explored in terms of increasing their reach, viability and arc of rotation. An attempt is made to refine the technique of flap harvestation to optimize outcomes of this versatile flap.

**METHODS:**

Six patients of complex lower limb defects were managed using the GMC flaps. Harvesting of the flap was always initiated from the posterior midline to include the proximal sural pedicle, sural nerve, short saphenous vein and the muscle belly of either the medial or the lateral gastrocnemius muscle along with the cutaneous paddle. All the flaps were islanded and denervated. The origin of the gastrocnemius muscle was detached in all cases to increase the reach of this flap.

**RESULTS:**

The flap can reliably and comfortably cover defects from middle third-lower third junction of thigh and the entire posterior aspect of the thigh. Such a local option offers relatively simple but more cost-effective approach to complex clinical problem with tolerable impairment of the donor site.

**CONCLUSION:**

The GMC flap can be considered as a worthwhile alternative to free-tissue transfer for limb salvage.

## INTRODUCTION

The gastrocnemius flaps are one of the most versatile and useful flaps in lower extremity reconstruction.^1^ Conventionally, they can be harvested as the proximally based medial gastrocnemius muscle or musculocutaneous flaps, proximally based lateral gastrocnemius muscle or musculocutaneous flaps for coverage of the knee and upper third tibia defects.^2^ They can also be harvested as distally based flaps based on branches of posterior tibial artery for lower third limb reconstruction.^3^^,^^4^ Flaps based on the perforators of the gastrocnemius muscle like the medial sural artery perforator flaps very commonly and the lateral sural artery perforator flaps rarely are gaining popularity in the recent days because of their thinness, and their ability to be used as pedicled or free flaps.^5^^,^^6^

Free functioning muscle transfer of the gastrocnemius musculocutaneous (GMC) flaps has also been reported to restore finger flexion of forearm in Volkmann’s contractures.^7^ Existing literature documents the use of the GMC flaps for coverage of defects from middle third of the leg to lower third of the thigh.^8^ We have used the GMC flaps for defects up to 20 cms above the knee joint with positive outcomes. This article is an attempt to refine the technique of flap harvestation thereby increasing the reach, reliability and arc of rotation of this flap.

## MATERIALS AND METHODS

A retrospective analysis of six cases of the GMC flap is presented. All these flaps were performed by the first author using the same technique. The details of the flap harvestation technique, defect dimensions, patient demographics and the flap details are presented. Regarding the surgical technique, the position of flap harvest; it is most comfortably done with the patient in prone position. However, the best compromise is supine position with the knee flexed and the hip abducted, flexed and externally rotated for a medial flap, and lateral decubitus position for a lateral flap.

Conventionally, it has been accepted that the medial GMC flap by virtue of its larger belly has the ability to support large skin paddle, and thereby has a longer reach. The number of musculocutaneous perforators traversing the medial belly is also more in number as compared to the lateral belly of the gastrocnemius muscle, thereby increasing its vascularity. The common peroneal nerve laterally is prone to injury and also has a tendency to limit the extent of the lateral GMC flap. Generally medial defects are covered by medial GMC flap and lateral defects by the lateral GMC flap. But in cases where the defects are higher up on the thigh, using a contra lateral GMC flap minimizes the twist on the pedicle by some degrees and may be preferred. Planning is done in reverse, and the skin island is marked in the posterior calf. The distal extent of the skin island can be extended up to 5 cms proximal to the malleoli for both the medial and lateral GMC flaps. Once the skin island is marked, a tail is extended from the skin island to the popliteal crease.

Planning of the tail by far is the most important step. The tail is extended in such a fashion so as to incorporate (i) The skin overlying either the medial or the lateral gastrocnemius belly, depending upon which flap is selected, (ii) The proximal cutaneous sural pedicle (Median, medial or lateral sural artery depending on presence in that particular case) and the lesser saphenous vein in the flap, and (iii) Most of the cutaneous perforators of the muscle bellies.

 Most of the perforators of the medial GMC flap are located 7 to 18 cms from the popliteal crease and at or within 1cm from a midline traced on the medial gastrocnemius muscle.^1^ Various authors have studied the location and branching patterns of the perforators of the medial and lateral sural arteries, and can be considered during planning of the skin paddle.^9^^,^^10^

The inclusion of the tail ascertains that all the perforators of the gastrocnemius muscle entering the skin are harvested along with the flap. Flap harvest is typically started from the posterior midline of the leg. As the skin incision is complete, the sural nerve and the short saphenous vein forms the landmarks for identifying the midline between the two gastrocnemius bellies. The sural nerve, short saphenous vein and the proximal cutaneous sural artery (Median, medial or lateral sural artery depending on presence in that particular case) are included in the flap. The skin and the fascia are sutured to each other so as to prevent damage to the vascular connections. By using combination of sharp and blunt dissection, the plane between both the gastrocnemius muscles and the gastrocnemius and the soleus is identified and developed with a finger. 

A finger is inserted beneath the gastrocnemius muscle and above the soleus muscle to include the entire gastrocnemius belly over the finger. The second incision is made over the lateral border of the muscle. The flap harvest is completed, by committing to the distal incision at last. Especially while harvesting the lateral GMF, the common peroneal nerve is safe guarded near the head of the fibula. Utmost care is taken to prevent damage to the paratenon over the tendoachillis during flap harvest. This flap now has the cutaneous sural artery, the sural vein, the short saphenous vein and all the musculocutaneous perforators from the gastrocnemius entering into the skin. 

**Fig. 1:**
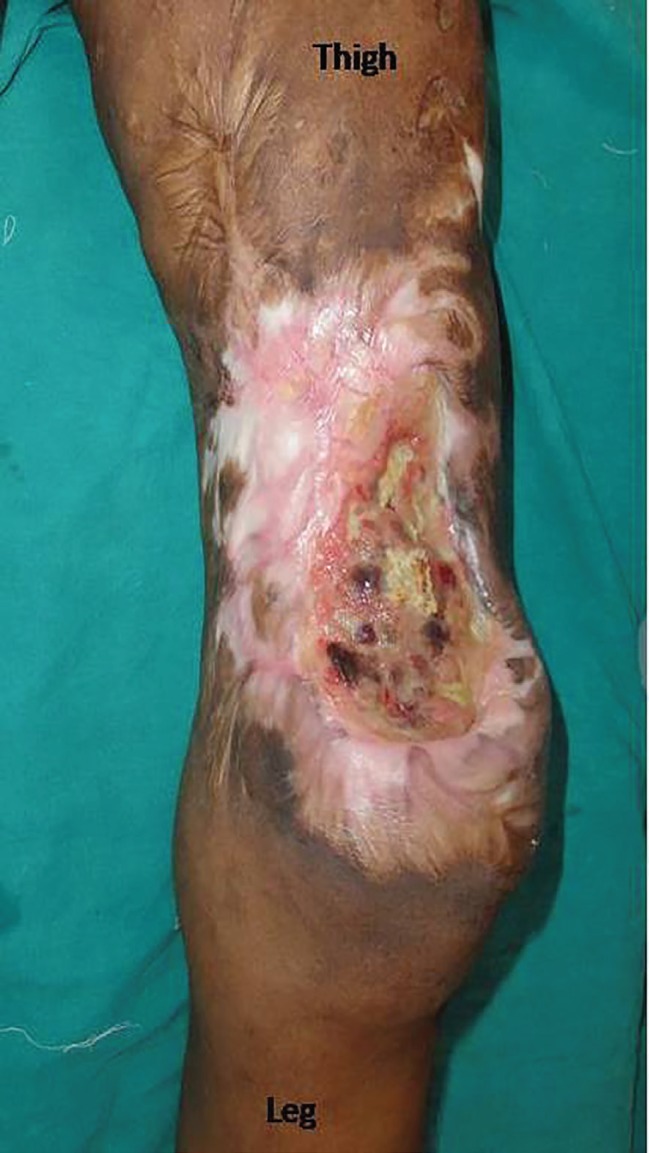
Defect on lower third thigh

**Fig. 2:**
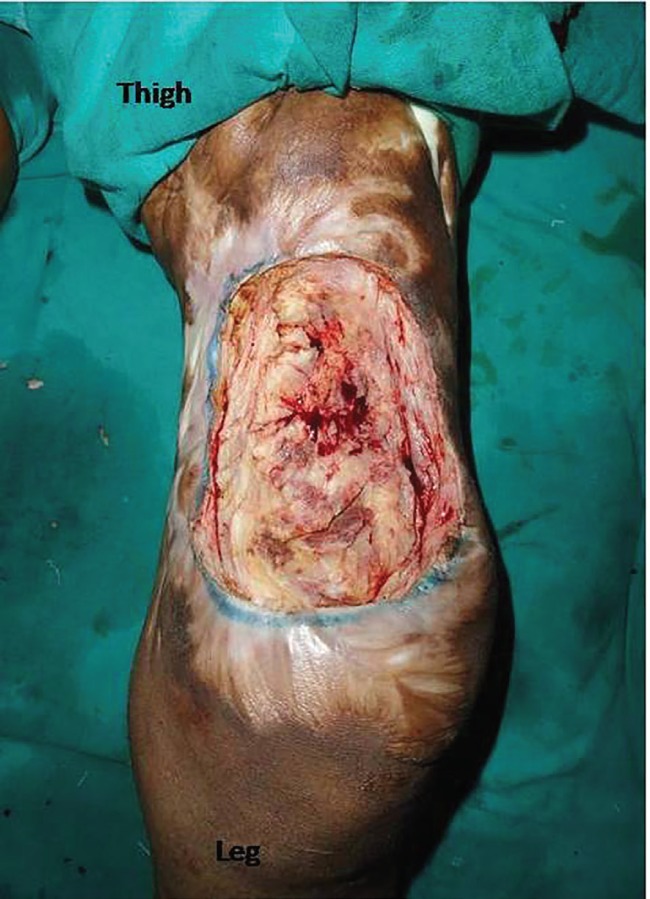
Post-debridement defect

**Fig. 3:**
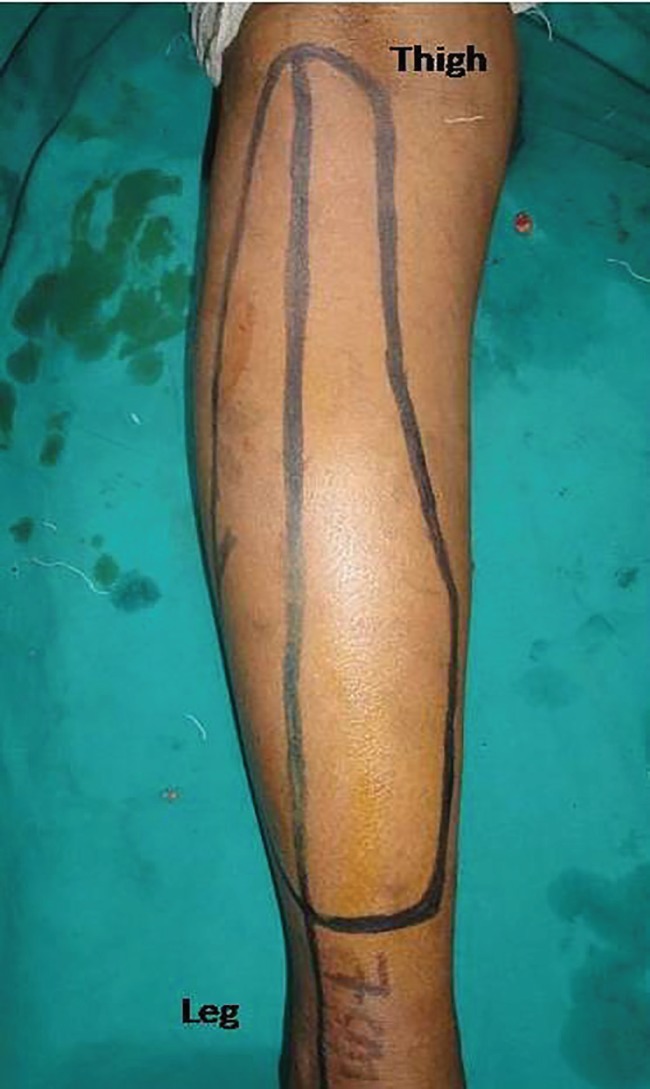
Markings of the GMC flap

**Fig. 4:**
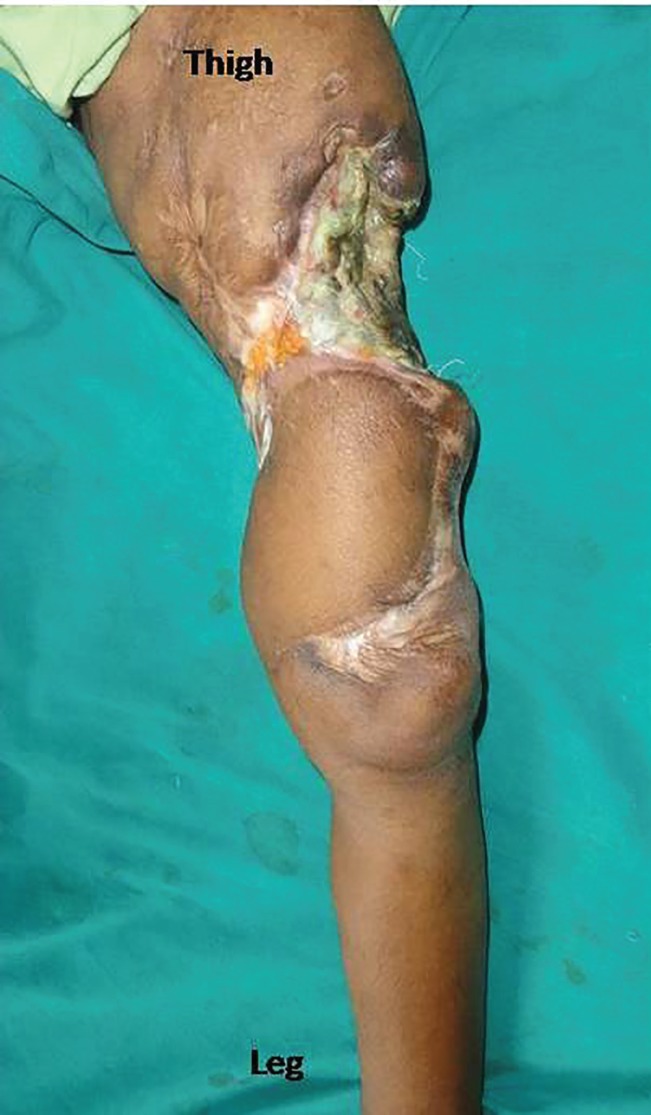
Well settled flap with new proximal defect

**Fig. 5:**
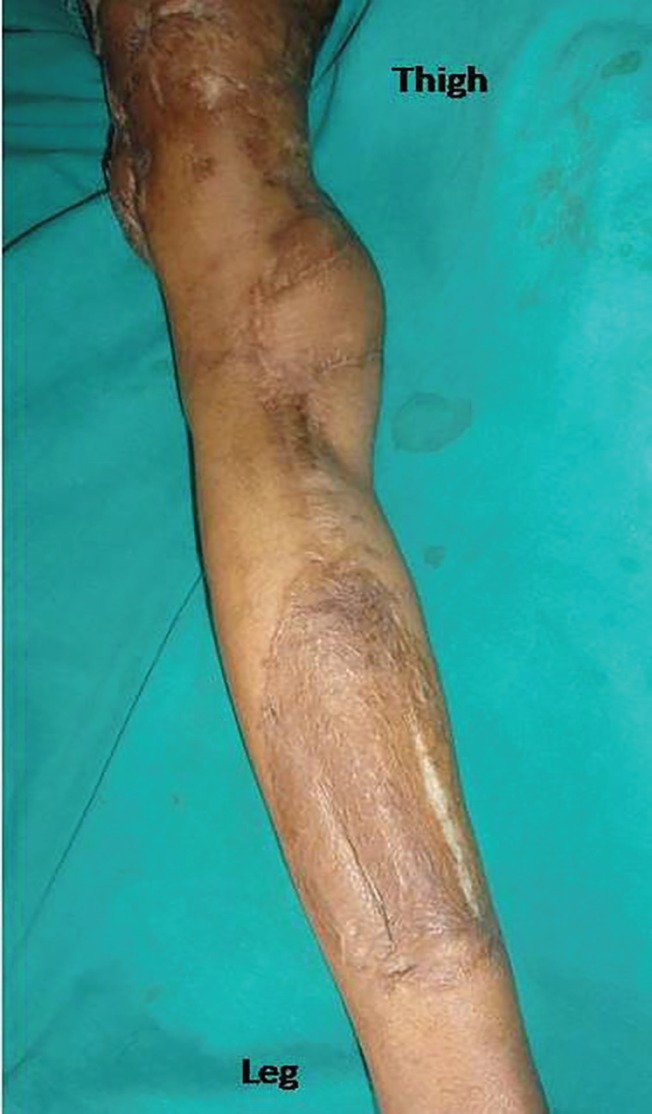
Well settled donor site

**Fig. 6:**
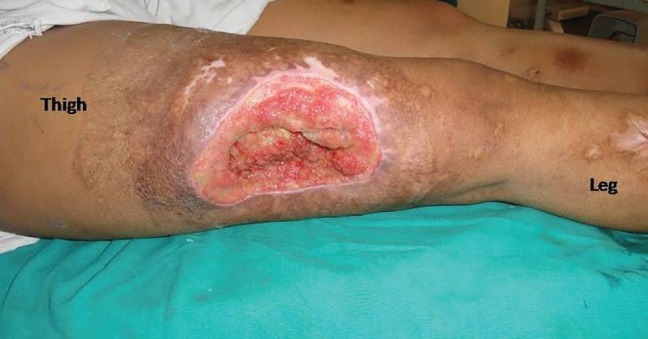
Middle third thigh defect

**Fig. 7:**
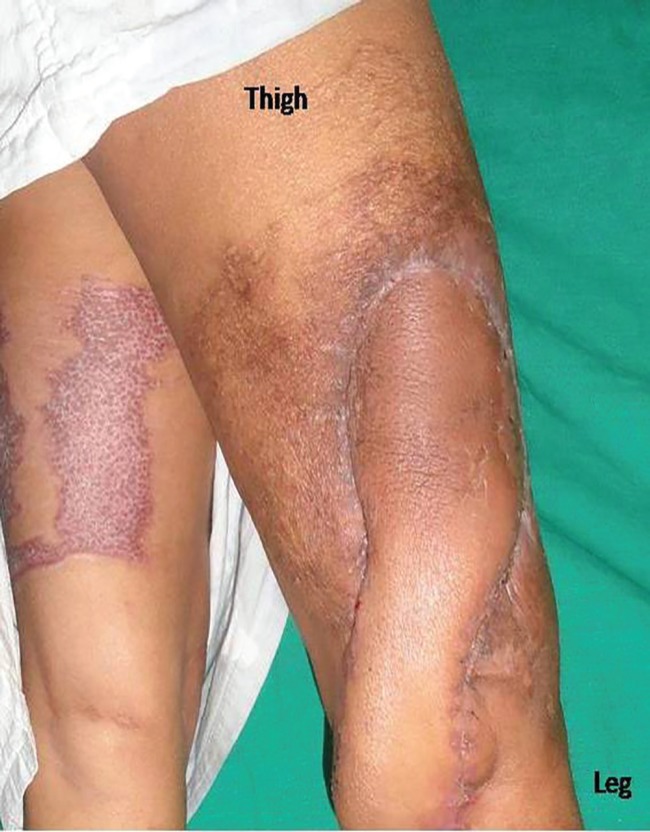
Well settled GMC flap with reach of 20 cm above knee joint

The motor nerve supplying the gastrocnemius muscle is identified and transected. The origin of the gastrocnemius muscle from the femoral condyle is identified and transected. The flap is islanded, taking due care to prevent trauma to the cutaneous sural pedicle in the popliteal crease. The pivot point of the flap is at or just below the popliteal crease, where the pedicle of the gastrocnemius enters the muscle. The intervening skin between the defect and the flap if any is incised and inset given. The donor defect is skin grafted. Few clinical cases are presented (Figure 1-7).

## RESULTS

The GMC flap was executed successfully in six cases over a period of six years and seven months from November 2008 to June 2016. The follow up ranged from 3 weeks to two years. Of the six patients, two were defects resulting from repeated fortwin injections due to addiction, one was due to burns and rests were because of trauma. The medial GMC flap was done in 4 patients, and the lateral GMC was done in 2 patients. The maximum width of the defect in our series was 10 cm, and the maximum length of the defect was 18 cms. The highest reach of the flap above the patella was 20 cms. All the flaps did well. There was delayed healing of the donor site in one patient, which healed with conservative management over a period of 6 weeks. There was a significant donor site aesthetic and contour deformity in all the patients. Details of patient demographics and flap are provided in Table 1.

**Table 1:** Details of patient demographics and flap

**No**	**Age**	**Sex**	**Mode of injury**	**Defect size**	**Distal most extent of defect from superior border of patella/above ankle joint**	**Flap used**
1	54	F	Burns	17 x 7 cm	15 (Above patella)	Lateral GMC
2	42	F	Fortwin Injections	15x10 cm	17 (Above patella)	Medial GMC
3	51	F	Trauma	17x15 cm	20 (Above patella)	Medial GMC
4	30	M	Trauma	14 x 8 cm	13 (Above patella)	Lateral GMC
5	36	M	Trauma	13x7 cm	14 (Above patella)	Medial GMC
6	45	F	Fortwin Injections	15x8 cm	22 (Above patella)	Medial GMC

GMC: Gastrocnemius musculocutaneous, M: Male, F: Female.

## DISCUSSION

GMC flap is not a very popular flap, mainly because of its poor donor site aesthetics. But there might be situations where in local tissue is not available for coverage in the thigh, or free tissue transfer may not be an option due to various reasons. It is under these circumstances that the GMC flap comes in very handy. McGraw *et al.*^11^ described the use of GMC flap for coverage of the upper tibia, knee joint and popliteal fossa. The range of rotation of this flap as demonstrated by the authors enabled coverage of small complex wounds to these regions. Sanders *et al.*^12^ used the GMC flap in a series of 8 cases for coverage of exposed prosthesis. Five were based on the medial head, and three were based on the lateral head. They felt that coverage of the joint with the flap helped in combating infection and providing a stable and reliable skin cover. They felt that the medial GMC flap could be raised within 5 cm of the medial malleoli, and the lateral GMC flap could be raised within 10 cms of the lateral malleoli. 

We feel that irrespective of the medial or the lateral gastrocnemius, both the flaps can be raised to within 5 cms of the malleoli because of the pattern of the underlying muscle and more number of vascular channels to nourish the flap as described in the technique. Gastrocnemius muscle being a type I muscle, the venous drainage in the muscles mirrors that of the arterial side.^13^ When arterial territories are linked by choke arteries or true anastomotic channels without changing caliber, the venous territories of the muscle, which drain in opposite direction are linked by avalvular oscillating veins. Therefore, the venous return is mainly towards the predominant arterial pedicle in the given territory.^13^

This minimizes the chances of venous congestion in this flap and gives reasonable safety in the harvest of distal skin paddles over the gastrocnemius muscle. Malawar and Price^14^ reported a series of 10 cases where gastrocnemius flaps were used to cover limb defects after tumour excision. The authors felt that the medial belly was more suitable for the distal femur and the proximal tibial defects, and the lateral belly was more suitable for the lateral fibular defects. Agarwal *et al.*^15^ reported the use of lateral GMC flap for coverage of femur on the lateral aspect of the thigh. They were able to cover the defect 14cms above the patella on the lateral aspect of the knee. They considered two delays before actually transferring the flap. It is interesting to note that they included the sural artery in the flap, and yet considered doing two delays of the GMC flap.

We feel that the delay procedure is unwarranted as it is a very robust flap, and can reach up to 20 cms above the patella without any delay procedure. Selecting a regional flap to cover the defect rather than free flaps which are higher up on the reconstructive ladder can be a better option, especially where the infrastructure and expertise for microvascular surgery is not available. It can also be considered as an option in low volume microsurgical centers when there is significant post traumatic vessel disease which precludes the use of the free flap or make it technically more demanding. There are certain modifications which we have done in the process of flap harvest, which we believe have influenced the outcomes in a positive way.

Conventionally, the sural nerve is excluded from the flap harvest. We have included the sural nerve in the flap harvest along with the sural artery and the short saphenous vein. This flap now has multiple blood supplies, namely: (i) Perforators of the gastrocnemius muscle entering the skin thought the course of the gastrocnemius muscle harvested, (ii) The proximal sural cutaneous pedicle (Median, medial or lateral sural artery depending on presence in that particular case), (iii) Neurocutaneous perforators of the sural nerve, and (iv) Venocutaneous perforators of the short saphenous vein.

This increases the vascularity of the flap significantly. In addition, we make it a point to transect the nerve to the muscle belly of the flap being harvested. This serves multiple purposes. First, it increases the arc of rotation of the flap by some degrees by neutralizing the tethering effect the nerve has on the flap. Second, the transaction of the nerve leads to atrophy of the flap in the long run, essentially serving as a debulking procedure and optimizing aesthetic outcomes.^16 ^Third, it relieves the patient of the potential painful spasms due to muscle contractions in the post operative period, and may reduce the chances of wound breakdown.^16^ Fourth, it has been proved experimentally that denervation can be a useful means of augmenting the vascularity of the flap.^17^^,^^18^

In a clinical setting, very logically transaction of the nerve, decreases the contractions of the muscle, thereby might reduce its nutritional requirements, and augmenting its blood supply. In those defects, which are further away from the knee joint, only the cutaneous component of the flap covers the defect. The muscle in these instances merely acts as a carrier of the vascular pedicles. In such cases it becomes even more important to transect the nerve so as to cause muscle atrophy and prevent bulging in the intervening area between the pivot point of the flap and the defect.

The distal reach of the flap in our series have been from 13 to 20 cms above the knee joint. This is almost more than 10 cms than that described in literature. We believe that the maneuvers which assisted us in increasing the reach of the flap were: 1) Including longer skin paddle by virtue of multiple blood supplies in the flap as mentioned above, 2): Transecting the nerve, 3): Islanding the flap, and 4): Transecting the origin of the muscle from the femoral condyle. The donor site of the GMC flap requires some serious mention. 

It not only leads to a contour deformity, but also leaves a visible skin graft with the contour deformity over the posterior aspect of the leg. If the paratenon is breached during flap harvest, the graft take is absent in that area. This can lead to delayed wound healing. The donor defect must be considered as an important parameter while making the decision of executing this flap, and must be discussed before hand with the patient. To minimize the donor defect, a medial sural artery perforator flap or a lateral sural artery perforator flap can be considered as a reconstructive option.

The GMC flap is a very versatile flap and can be used to resurface large defects from the middle third-lower third leg junction to middle third-lower third junction of the thigh. The cosmetic outcome of the donor site is unfavorable. This flap may be used in select cases for coverage of complex wounds, especially when free tissue transfer is not an option. Such a loco-regional option offers relatively simple but more cost-effective approach to complex clinical problem with tolerable impairment of the donor site and should be considered as a worthwhile alternative to free-tissue transfer for limb salvage.

## CONFLICT OF INTEREST

The authors declare no conflict of interest.
